# YAP is required for prostate development, regeneration, and prostate stem cell function

**DOI:** 10.1038/s41420-023-01637-1

**Published:** 2023-09-09

**Authors:** Hui Xie, Linpei Guo, Qianwang Ma, Wenyi Zhang, Zhao Yang, Zhun Wang, Shuanghe Peng, Keruo Wang, Simeng Wen, Zhiqun Shang, Yuanjie Niu

**Affiliations:** 1 Department of Urology, Tianjin Institute of Urology, The second hospital of Tianjin Medical University, 300211 Tianjin, China; 2 Gene and Immunotherapy Center, The Second Hospital of Shandong University, 250033 Jinan, Shandong China; 3 Department of Radiology, The second hospital of Tianjin Medical University, 300211 Tianjin, China; 4 Department of Pathology, Tianjin Institute of Urology, The second hospital of Tianjin Medical University, 300211 Tianjin, China

**Keywords:** Regeneration, Stem cells

## Abstract

Prostate development and regeneration depend on prostate stem cell function, the delicate balance of stem cell self-renewal and differentiation. However, mechanisms modulating prostate stem cell function remain poorly identified. Here, we explored the roles of Yes-associated protein 1 (YAP) in prostate stem cells, prostate development and regeneration. Using YAP^fl/fl^, CD133-CreER mice, we found that stem cell-specific YAP-deficient mice had compromised branching morphogenesis and epithelial differentiation, resulting in damaged prostate development. YAP inhibition also significantly affected the regeneration process of mice prostate, leading to impaired regenerated prostate. Furthermore, YAP ablation in prostate stem cells significantly reduced its self-renewal activity in vitro, and attenuated prostate regeneration of prostate grafts in vivo. Further analysis revealed a decrease in Notch and Hedgehog pathways expression in YAP inhibition cells, and treatment with exogenous Shh partially restored the self-renewal ability of prostate sphere cells. Taken together, our results revealed the roles of YAP in prostate stem cell function and prostate development and regeneration through regulation of the Notch and Hedgehog signaling pathways.

## Introduction

Prostate cancer has become one of the most common and lethal diseases among American men [1]. Although most prostate cancer cases show an indolent tumor behavior and prostate cancer treatment is developing rapidly, it remains a major public health problem. Therefore, the malignant disease has been the focus of intense investigation to understand its pathobiology and provide improved treatment [2, 3]. Though the hypothesis that malignancy, including prostate cancer, may be aroused due to a re-awakening of the developmental process that occur during organogenesis has not been fully proved, several recent researches have demonstrated key similarities in gene expression patterns between prostate organogenesis and cancer [4–6]. Thus, to gain a deeper understanding of prostate development is considerably rational.

The most distinctive features of stem cell are self-renewal and differentiation, and the delicate balance between them drives organ development and repair [7, 8]. Therefore, the deregulation of these biological process leads to cancer and developmental defects. However, many of the critical regulators of stem cell and the underlying mechanisms remain poorly identified. The prostate is a small walnut-sized male sex accessory gland that is located below the bladder and surrounds the urethra. In the mouse, the initial outgrowth of epithelial buds arises at approximately E17.5 days in a 19–21-day gestation strain [9]. The continuous morphogenesis process can be categorized in a series of developmental stages, including organ determination, epithelial initiation or budding, branching morphogenesis, differentiation, and pubertal maturation [10]. In the neonatal prostate, stem cells differentiate into basal, luminal and neuroendocrine cells and contribute to the development of the prostate [11]. In contrast, in the adult prostate, the respective stem/progenitor cells within the lumina and basal cell lineages are relatively quiescent and maintain epithelial homeostasis [12]. Meanwhile, many researches have shown that stem cells within basal cell lineage retain the capacity for multi-lineage differentiation [13, 14]. Although many efforts have been made in the study of prostate stem cell self-renewal and differentiation, underlying mechanism of prostate stem cell regulation remains largely unknown [15–18].

As the downstream effector of the Hippo pathway, the Yes-associated protein (YAP, also known as YAP1) is a multifunctional transcriptional coactivator acting by binding to the TEA domain family members (TEAD), and plays crucial roles in organ size regulation [19, 20]. Several studies have reported that mutations of Hippo pathway kinase or YAP overexpression leads to the overgrowth of various organ and appendages [20–23]. Targeting deletion of YAP^-/-^ in mice results in developmental arrest around E8.5d, demonstrating a critical role for normal development [24]. Meanwhile, YAP also take a crucial role in maintaining stem cell pluripotency. It has been reported that YAP knockdown leads to loss of embryonic stem (ES) cell pluripotency, while ectopic expression of YAP prevents ES cell differentiation in vitro and maintains stem cell phenotypes even under differentiation conditions [25]. Moreover, YAP regulates a multitude of stem cell-related genes, including Nanog、Oct4 and Sox2, via binding directly to their promoters [25, 26]. Thus, it was reasonable to expect that YAP could function as a key stem cell regulator and be critical for prostate development. Here, we determined the role of YAP in the functional regulation of prostate stem cell by applying time- and organ-conditional-specific YAP-deficient transgenic mouse model, prostate regeneration model, and primary prostate sphere culture.

## Results

### Generation and characterization of stem cell-specific YAP knockout (scYAPKO) mice

**Fig. 1:**
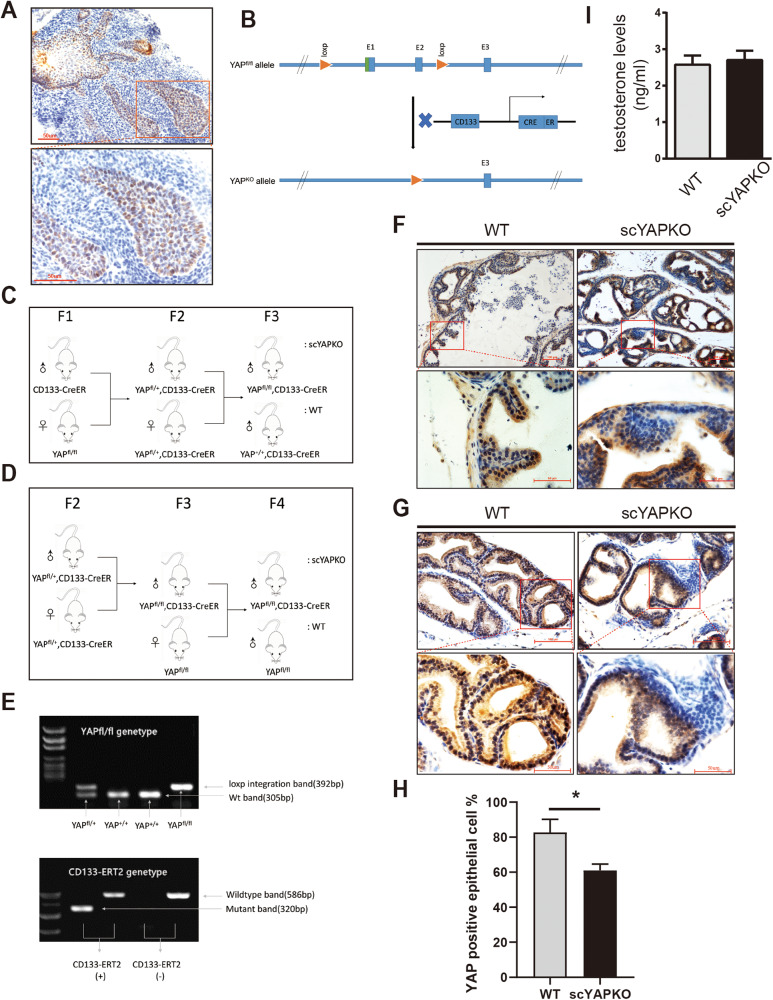
Generation and genotyping of scYAPKO mice by Cre-loxP strategy. **A** The expression of YAP in the urogenital sinus of E0 mice was investigated via IHC. Scale bar is 50 µm. **B** Schematic of the floxed YAP alleles for conditional disruption. The genomic DNA containing exons 1–3 was shown and exons 1–2 were disrupted via CreER recombinase. **C**, **D** Breeding strategy to generate the tissue-specific scYAPKO mice. YAP^fl/fl^, CD133-CreER littermates were applied as scYAPKO mice and YAP^fl/fl^ mice or YAP^+/+^, CD133-CreER littermates were used as WT controls. **E** Genotypes of tail snips of YAP^fl/fl^, CD133-CreER mice. The CD133 gene promoter is selectively expressed in the stem cells. The size of floxed YAP, WT YAP, CD133-ERT2 mutant band, and WT band are 392 bp, 305 bp, 320 bp, and 586 bp, respectively. **F**, **G** The expression of YAP in 6-week-old scYAPKO and WT APs and DLPs were determined by IHC, respectively, scale bars are shown. The quantification data was quantified and presented in (**H**). **I** The serum T levels in the adult WT and scYAPKO males. Results are presented as mean ± SEM (*n* = 4). IHC immunohistochemistry, scYAPKO stem cell-specific YAP knockout, WT wild-type, AP anterior prostate, DLP dorsal–lateral prostate, T testosterone.

To explore the function of YAP in prostate stem cells, which play key roles in prostate development, and avoid lethality of YAP deletion embryo, the Loxp-CreER recombination system was applied to conditionally inactivate the YAP alleles in stem cells of the urogenital sinus at late embryonic stages (E17) by Tamoxifen induction. As Fig. 1A depicted, YAP was highly expressed in urogenital sinus-enriched prostate stem cell. CD133, a five-transmembrane domain containing glycoprotein, is expressed on the surface of a variety of normal stem cell and cancer stem cell, including prostate [27–31]. Combining flox YAP mice with CreER recombinase linked to the CD133 promoter, we were able to develop stem cell-specific YAP knockout (scYAPKO) mice (Fig. 1B–D). For experimental purposes, YAP^fl/fl^ mice or YAP^+/+^, CD133-CreER littermates were used as wild-type (WT) controls. Genotyping results from both groups are demonstrated in Fig. 1E.

To confirm that YAP proteins have been partially deleted in YAPKO mice prostate, YAP immunohistochemistry (IHC) staining was applied (Fig. 1F, G). We found that YAP expression was significantly reduced in scYAPKO mice compared to WT mice, and cells with reduced YAP expression showed a regional distribution (Fig. 1F–H). Moreover, to ensure that deletion of stem cell YAP will not affect serum testosterone concentration, the serum from scYAPKO and WT mice was collected and we found no obvious difference in testosterone concentration (Fig. 1I). In conclusion, we successfully developed a conditional specific YAP-deficient transgenic mouse model and validated its efficacy.

### Reduced prostate size with impaired branching morphogenesis and partial loss of glandular epithelial infolding in scYAPKO mouse prostate

**Fig. 2:**
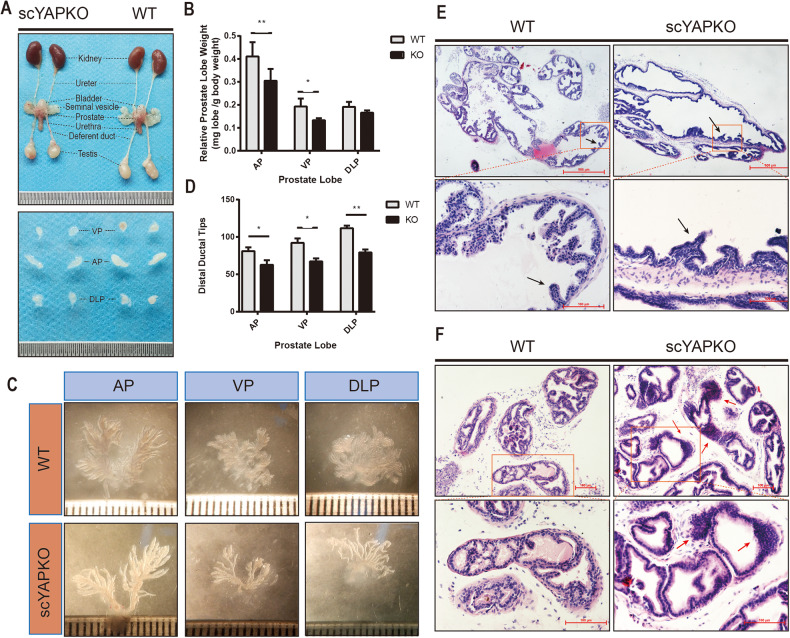
Disruption of YAP alleles on stem cells leads to perturbed prostate morphogenesis and histology. **A** Overview of the prostate glands dissected from 6-week-old WT mice and scYAPKO mice. **B** Relative prostate lobe weight (mg lobe/g body weight) of scYAPKO mice were measured. **C** Epithelial ducts were dissected from each prostate lobes of the prostate of 6-week-old mice from both genotypes, and the quantification data are shown in (**D**). The data shown above are presented as mean ± SEM (*P < 0.05 vs. WT littermate controls; **P < 0.01 vs. WT littermate controls, *n* = 4). **E**, **F** Histologic examination of 6-week-old WT and scYAPKO mice prostates. AP and DLP tissue sections were subjected to hematoxylin and eosin staining. The infolding glandular epithelium is shown by black arrows. The irregular region is shown by red arrows. Scale bars are shown. *n* = 4–5 for each group. AP anterior prostate, DLP dorsal–lateral prostate, VP ventral prostate.

To examine the phenotype changes between two groups after prostate development, mice were sacrificed at 6 weeks old when the prostate has gone through most of the developmental process and is basically mature. The gross appearance from 6-week-old scYAPKO anterior prostate (AP) and ventral prostate (VP) exhibited slight size reduction as compared with WT; however, dorsal–lateral prostate (DLP) was comparable (Fig. 2A). Then we quantified the scale of prostate lobes using relative prostate lobe weight (mg lobe/g body weight). We found that the weight of scYAPKO AP and VP were reduced compared to WT AP and VP while DLP from both groups were comparable (Fig. 2B). During ductal morphogenesis, the prostatic epithelial buds elongate and canalize to form ducts, which ultimately give rise to paired lobes with unique branching patterns [32]. To analyze the branching morphogenesis, microdissection of digested prostate lobes from WT and scYAPKO mouse were applied (Fig. 2C). The results showed a significant reduction in branching ducts in all scYAPKO prostate lobes. The quantification results of ductal tips were also provided (Fig. 2D). Meanwhile, the dilated and malformed prostatic ducts were also found in scYAPKO mice, which may explain the slight difference in prostate size and weight between the two groups.

The histological examinations were also carried out to show histological differences (Fig. 2E, F). The prostate epithelial cells often form infolding within each duct, thereby increasing surface area [33]. Compared with WT prostates, scYAPKO prostates performed a distinct decrease of the infolding structure (Fig. 2E, quantitative results depicted in Supplementary Fig. 1). In addition, there were several regions composed of irregularly arranged cells in scYAPKO prostate sections (as depicted by red arrows in Fig. 2F). While WT luminal cells aligned and formed a single layer, scYAPKO luminal cells often aggregated as clusters, possibly due to impaired cell migration. Taken together, our data suggested that stem cell YAP plays critical roles in prostate morphogenesis and development.

### YAP ablation regulates prostate epithelial cell proliferation, apoptosis, and impairs luminal epithelial cell maturation

**Fig. 3:**
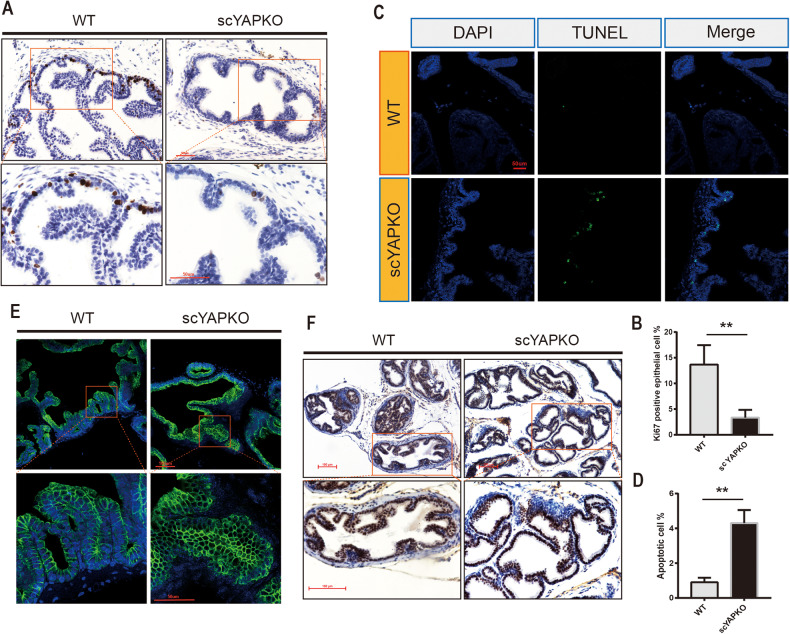
Impaired proliferation, apoptosis, epithelial differentiation, and function in scYAPKO prostate. **A** The proliferative index of DLP from both genotypes was determined by Ki67 IHC. Scale bar is 100 µm. The proliferative index is quantified and presented in (**B**). The data are presented as mean ± SEM (*P < 0.05 vs. WT littermate controls; **P < 0.01 vs. WT littermate controls, *n* = 4). **C** Terminal deoxynucleotide transferase-mediated dUTP nick end-labeling (TUNEL) assay showed increased apoptosis signals (green) in scYAPKO mice. The green fluorescence indicated apoptotic cells and 4’, 6-diamidino-2-phenylindole counterstaining served as nuclei labeling. Scale bar is shown. The quantification data was quantified and presented in (**D**). The data are presented as mean ± SEM (*P < 0.05 vs. WT littermate controls; **P < 0.01 vs. WT littermate controls, *n* = 4). **E** CK8 immunofluorescence in DLP of 6-week-old mice from both genotypes. Scale bars are shown. **F** AR IHC in the adult scYAPKO and WT littermate prostates. Scale bar is 100um.

To explore the possible mechanisms leading to impaired prostate development of the scYAPKO mice, we applied Ki67 immunohistochemistry and TUNEL assay to evaluate the proliferation and apoptosis of prostate epithelial cells. The wild-type mouse prostate undergoes extensive branching morphogenesis and 85% of the adult number of ductal tips and branch points are formed during the first 2 weeks after birth [9]. By puberty, when prostate start to mature in response to androgen, the proliferation of epithelial cells rised again [15, 33]. Therefore, we compared the proliferative nuclei staining from 4-week-old scYAPKO and WT prostates. The results indicated that scYAPKO prostate displayed less proliferative epithelial cells compared with WT controls, possibly leading to diminished branching morphogenesis (Fig. 3A, B). To investigate apoptosis in prostate epithelial cells, we applied TUNEL assay on mice at 6 weeks of age. While WT prostates showed barely detectable apoptotic cells, we identified more apoptotic cells located in the lumen of scYAPKO mouse prostates (Fig. 3C, D). The increased apoptotic signals in scYAPKO mouse prostates indicated that YAP-mediated signaling plays an important role in maintaining epithelial cell survival.

In normal mouse prostate, functional luminal epithelial cells express cytokeratin 8 (CK8) and androgen receptor (AR) [33, 34]. We next examined the consequences of YAP loss on luminal cell. Immunofluorescence of CK8 and Immunohistochemistry of AR were applied. In WT mouse prostates of 6 weeks age, luminal cells were tall columnar epithelial cells aligned and formed a single layer. However, some regions in scYAPKO mouse prostates were composed of irregularly arranged cells (Figs. 2E, F and 3E). These luminal cells lacked columnar morphology, aggregated as clusters, had decreased cytoplasmic content and more crowded, condensed nuclei. Furthermore, as Fig. 3F shows, AR expression of these abnormal luminal cells in scYAPKO mouse prostates was significantly reduced compared with WT mouse. These results indicate that YAP ablation on stem cell impairs stem cell differentiation and luminal cell maturation. Together, these data imply that YAP expressed on stem cells promotes epithelial cell proliferation and branching morphogenesis in early prostate development, and may inhibit epithelial cell apoptosis and affect prostate stem cell differentiation during later development stages.

### YAP regulates prostate regeneration and branching morphogenesis

**Fig. 4:**
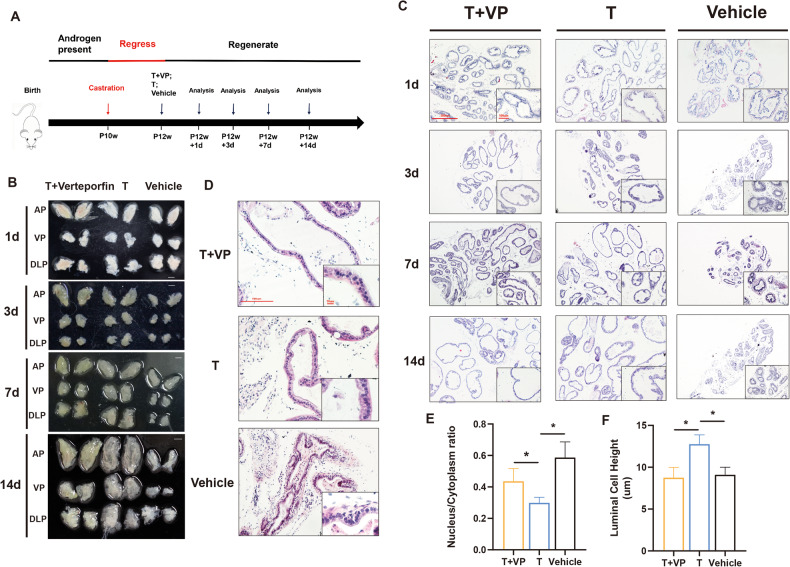
YAP inactivation impaired prostate regeneration in vivo. **A** Schematic figure showing the experimental strategy for prostate regeneration using YAP inhibitor Verteporfin (VP). **B** Overview of the prostates after regeneration at different time periods. *n* = 3 for each group, Scale bar 2 mm. **C** Hematoxylin & Eosin staining of ventral prostate sections at indicated regeneration stages. **D** Histologic examination of ventral prostate sections under oil lens after 14 days of regeneration. **E**, **F** The nucleus/cytoplasm ratio and luminal cell height of ventral prostates epithelial after 14 days of regeneration were quantified. Bar graph expresses mean ± SEM from three pairs of mice, five sections per prostate, *P* < 0.05. VP verteporfin.

As a male sex accessory gland, the sensitive response of the prostate to androgen makes it an ideal model for studying stem cell properties. It has long been recognized that the rodent prostate has an infinite cyclic regenerative capacity, atrophying in the presence of androgen deprivation but regenerating the organ in response to androgen replenishment [35]. Therefore, rodent prostate stem cell can be defined by their ability to regenerate the prostate during the androgen “deprivation-repletion” cycle. To explore the function of YAP in prostate regeneration, Verteporfin (VP), an inhibitor of YAP, was used to inhibit the function of YAP (Fig. 4A). As the regeneration process proceeded, YAP inhibition in mice resulted in a dramatic decrease in prostate size compared to normal regeneration, especially at day 14 (Fig. 4B). However, the relative prostate weight was similar in both groups (Supplementary Fig. 2). Next, the prostate histology at different stages of regeneration was compared (Fig. 4C and Supplementary Fig. 3). The vehicle group showed significantly regressed prostate histology at all stages of regeneration, such as dramatically regressed lumen and atrophied cytoplasm. As for the other two groups, several deficiencies could be found in YAP-inhibited prostate in addition to the decreased gland size. First, reduced luminal folding was noticeable in YAP-inhibited prostates beginning at day 3 (Fig. 4C). Second, by 14 days, normal regeneration prostate luminal cells aligned regularly and formed a monolayer with columnar luminal cells showing a natural nuclear versus cytoplasmic ratio, while YAP-inhibited prostate luminal cells lacked columnar morphology, had reduced cytoplasmic content and more crowed, condensed nuclei (Fig. 4D–F). Third, lumen secreta of YAP-inhibited prostate at day 14 was reduced compared to normal regeneration, implying to some extent a disruption of luminal cell function. Together, above results suggested that YAP plays critical roles in prostate regeneration and branching morphogenesis.

### YAP is required for prostate regeneration and epithelial cell differentiation

**Fig. 5:**
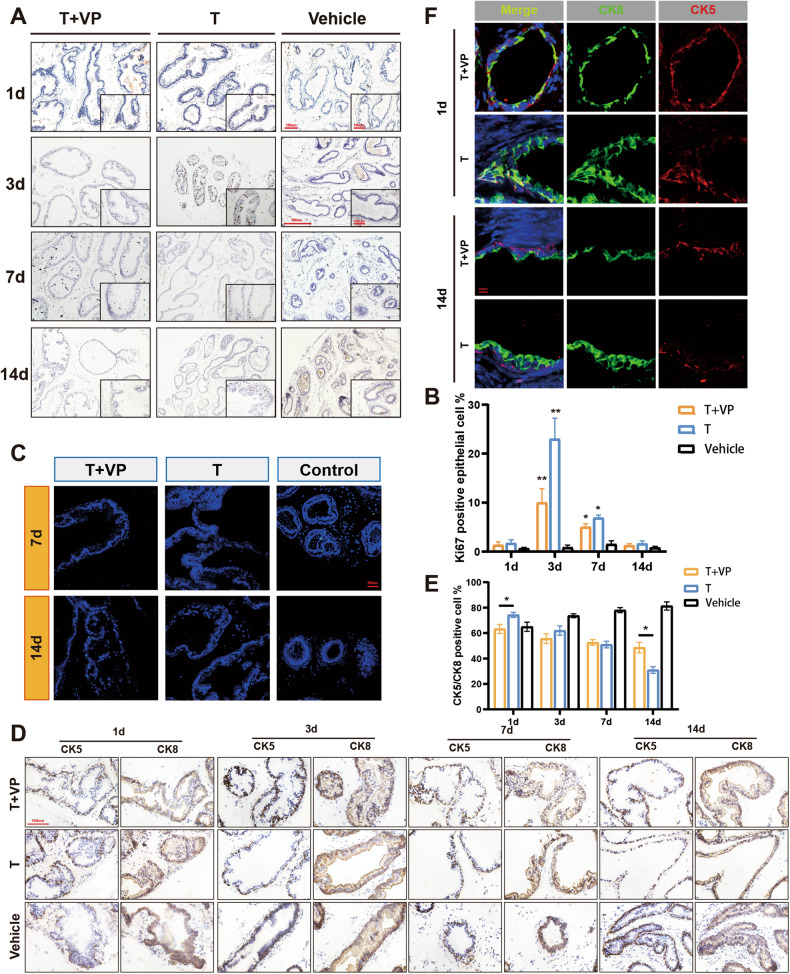
YAP ablation diminished epithelial proliferation and stem cell differentiation. **A** Ki67 immunohistochemistry staining shows proliferating cells in ventral prostate at indicated regeneration stages. **B** Quantification of Ki67-positive epithelial cells from three pairs of mice. The data are presented as mean ± SEM (*P < 0.05 **P < 0.01). **C** Apoptotic epithelial cells detected by TUNEL assay in prostates at late stages of regeneration (7d and 14d). **D** CK5 and CK8 immunohistochemistry staining in serial sections of ventral prostate at indicated regeneration stages. **E** Quantification of basal versus luminal epithelial cell ratios of ventral prostates at indicated regeneration stages. The data is presented as mean ± SEM (*P < 0.05). **F** Co-IF of CK5 (red) and CK8 (green) in regenerated prostates at indicated regeneration stages. VP verteporfin.

To uncover how YAP regulates prostate regeneration, we examined cell proliferation and apoptosis during prostate regeneration (Fig. 5A–C). In the vehicle group, the proliferation rate (% Ki67^+^) of regressed prostate epithelial cells remained consistently low because of the absence of androgen stimulation. In the other two groups, as the regeneration time progressed, the proliferation of epithelial cell first increased and then decreased, and the most vigorous proliferation was at 3d and 7d. By 14d, the epithelial cell proliferation rate was dramatically reduced. However, in 3d and 7d YAP-inhibited prostate, Ki67-positive cells were significantly reduced compared to normal regeneration prostate, possibly leading to a reduction in gland size (Fig. 5A, B). On the other hand, the apoptotic cells were significantly increased in the regressed prostate than in the other two groups. Moreover, we could find several apoptotic cells in YAP-inhibited prostate, especially at day 14, whereas no apoptotic cells were found in normal regeneration prostate (Fig. 5C).

Next, we investigated the consequences of YAP inhibition on basal and luminal cell layers (Fig. 5D–F). In the regressed prostate, the ratio of basal to luminal cells (% CK5^+^/CK8^+^ positive cell) was increasing due to the continued apoptosis of luminal cells. Meanwhile, the ratio of basal to luminal cells in normal regenerating prostates kept decreasing due to the differentiation of CK5^+^/CK8^+^ intermediate cells and the continued loss of CK5 expression. As for YAP-inhibited prostates, the ratio of basal to luminal cells also decreased. However, on day 1, the ratio of basal to luminal cells in YAP-inhibited prostates was significantly reduced compared to normal regenerating prostates, yet by day 14 it became higher than in normal regenerating prostates (Fig. 5D–F). Moreover, CK5^+^/CK8^+^ intermediate cells on day 1 were also decreased in YAP-inhibited prostates (Fig. 5F). Finally, YAP-inhibited prostates retained a high ratio of basal to luminal cells, suggesting that the differentiation of CK5^+^/CK8^+^ intermediate cells was arrested by YAP inactivation. Together, these data indicate a critical role of YAP in prostate cell fate determination.

### YAP regulates prostate regeneration via Notch signaling and Hedgehog signaling

**Fig. 6:**
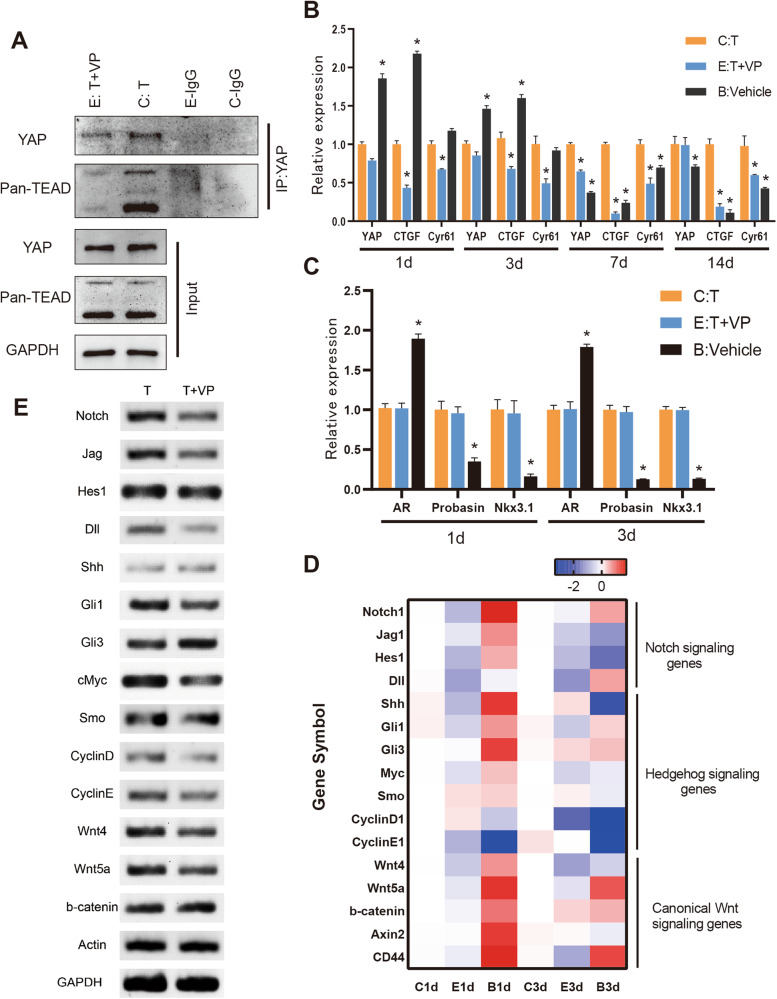
Identification of potential YAP-regulated signaling pathway in prostate development and regeneration. **A** Co-Immunoprecipitation detection of YAP/TEADs interactions in YAP-inhibited and normal regenerated prostate. **B** The expression of YAP, CTGF, and Cyr61 in regenerated prostates at indicated regeneration stages. **C** The expression of AR, Probasin, and Nkx3.1 in regenerated prostates at early stages of regeneration. **D** Heatmap of critical pathway genes associated with prostate stem cell in early prostate regeneration were compared. **E** RT-PCR detection of several critical pathway genes associated with prostate stem cell between YAP-inhibited and normal regenerated prostates in early prostate regeneration. VP verteporfin.

Given that verteporfin inhibits YAP function primarily by competitively binding to its downstream transcription factor TEADs (TEAD1-4) [19, 36, 37], we used Co-IP assay to verify that YAP function was suppressed. In YAP-inhibited prostates, a dramatic reduction in TEADs proteins bound to YAP was found compared to normal regenerating prostates (Fig. 6A). Next, we examined the mRNA expression of YAP and several key genes directly regulated by it in all groups. Although the expression of YAP was comparable in both YAP-inhibited and normally regenerating prostates, the expression of its direct targets, including CTGF and Cyr6, was dramatically decreased in YAP-inhibited prostates compared with normally regenerating prostates (Fig. 6B).

Since AR signaling is essential for the development and regeneration of the prostate, the mRNA expression of AR and its classical targets including Probasin and Nkx3.1 during early regeneration was first examined to explore the potential molecular mechanisms of YAP regulation. Unfortunately, we did not find significant differences between YAP-inhibited and normally regenerating prostates (Fig. 6C). Then we examined mRNA expression of several key genes and transcription factors critical for prostate development and regeneration during early regeneration (Fig. 6D, E). As shown in Fig. 6D, E, the expression of Notch signaling and Hedgehog signaling genes of YAP-inhibited prostates, including Notch1, Hes1, Dll, Shh, Gli1, cyclinD1, and cyclinE1, was significantly decreased compared to normal regenerating prostates. These results suggest that YAP may modulate prostate progenitor cell proliferation and differentiation through Notch signaling and Hedgehog signaling.

### YAP inactivation in prostate stem cells leads to impaired differentiation and in vivo prostate graft regeneration

**Fig. 7:**
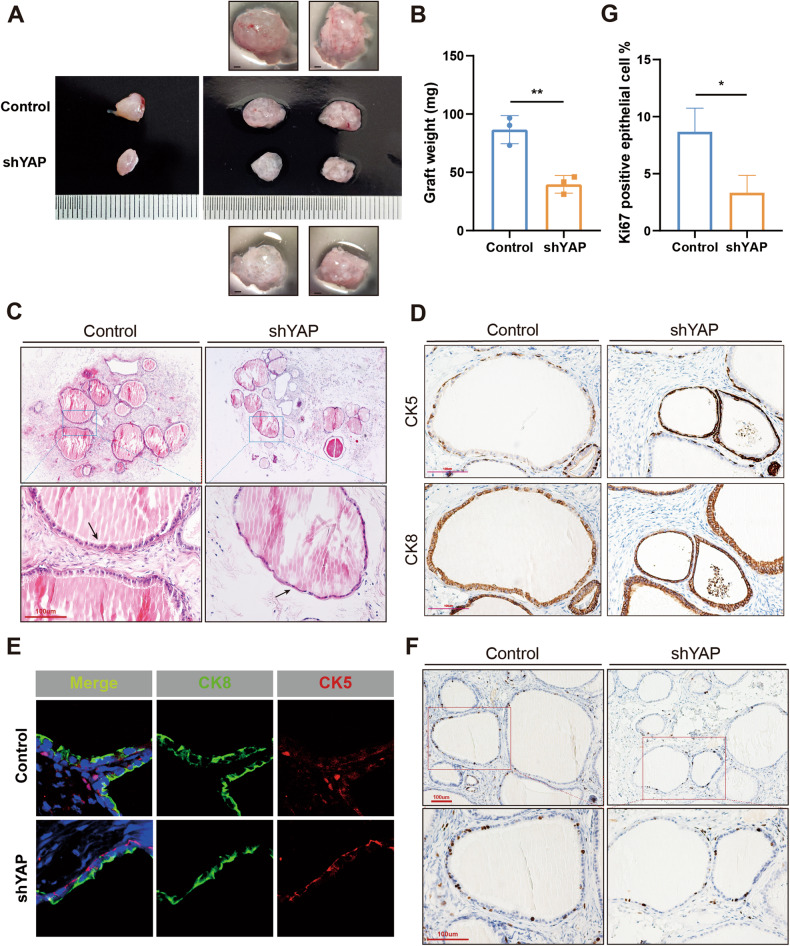
YAP inactivation in prostate stem cells leads to impaired differentiation and in vivo prostate graft regeneration. **A** The overview of shYAP and control prostatic grafts after 8 weeks of regeneration in vivo. **B** Prostatic wet weight was compared between shYAP and control groups. The data are presented as mean ± SEM (**P < 0.01, *n* = 3). **C** Hematoxylin & Eosin staining of regenerated prostatic grafts. **D** Ki67 IHC shows proliferating cells in regenerated prostatic grafts. **E** Quantification of Ki67-positive epithelial cells in both groups. The data are presented as mean ± SEM (*P < 0.05). **F** CK5 and CK8 immunohistochemistry staining in serial sections of regenerated prostatic grafts from both groups. **G** Co-IF of CK5 (red) and CK8 (green) in regenerated prostatic grafts.

To examine the function of YAP in prostate stem cell self-renewal and differentiation in vivo, and to eliminate possible mesenchymal and hormonal effects of YAP inactivation, the prostate graft regeneration assay was performed using shYAP or control prostate primary cells mixed with normal urogenital sinus mesenchymal (UGSM) cells (Supplementary Fig. 4). Two months after engraftment into the SCID mice, both shYAP and control prostate stem cells were able to regenerate glandular structures (Fig. 7A). However, shYAP prostatic grafts weight was dramatically reduced and the number of glandular structures was also significantly decreased (Fig. 7A, B). Moreover, the enfolding and epithelial morphology of glandular structure were compromised in shYAP grafts (Fig. 7C). Interestingly, examination of graft cell lineage commitment revealed the same pattern as depicted in prostate development and regeneration. In control grafts, the luminal cells which was completely differentiated exhibited a typical columnar secretory epithelial morphology and aligned in a single layer; in addition, only limited numbers of basal cells were present. In contrast, shYAP grafts lacked columnar morphology and had reduced cytoplasmic content, as well as the persistence of high number of basal cells (Fig. 7C–E). Furthermore, the epithelial proliferation rate (% Ki67^+^) of shYAP grafts was also significantly decreased compared to control grafts (Fig. 7F, G). Taken together, these data suggest that YAP functions in a stem cell–cell-autonomous manner to modulate prostate epithelial proliferation and differentiation, as well as branching morphogenesis.

### YAP regulates prostate stem cells through Notch signaling and Hedgehog signaling

**Fig. 8:**
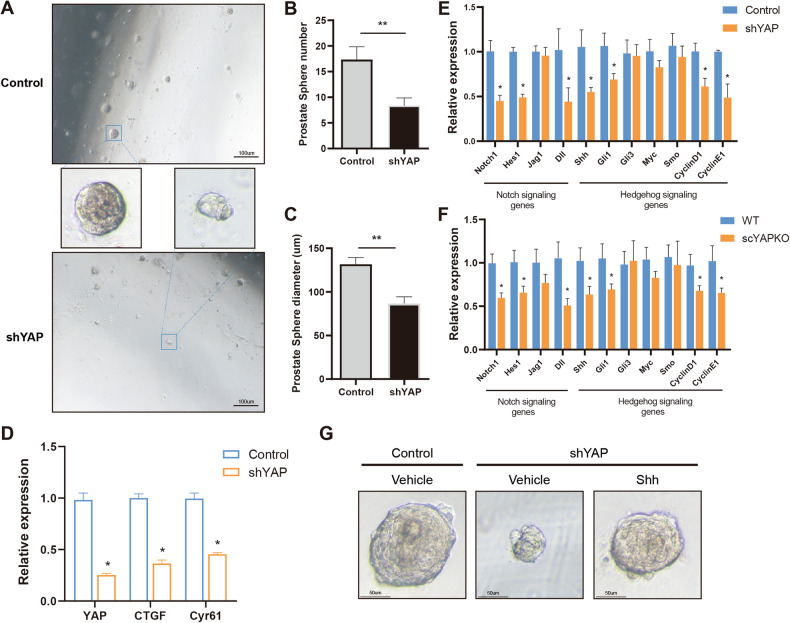
YAP regulates prostate stem cells through Notch signaling and Hedgehog signaling. **A** The morphology of shYAP and control prostate spheres cultured in Matrigel. **B** The number of prostate spheres were measured from 3 pairs of sphere samples. The data is presented as mean ± SEM (**P < 0.01). **C** The diameter of prostate spheres was measured in at least five prostate spheres from three pairs of sphere samples. The data are presented as mean ± SEM (**P < 0.01). **D** The expression of YAP, CTGF and Cyr61 in prostate spheres. **E** Notch and Hedgehog signaling pathways were compared by qPCR between shYAP and control prostate spheres. **F** Notch and Hedgehog signaling pathways in early prostate development were compared via qPCR between scYAPKO and WT in 2-week-old mouse prostates. **G** Prostate spheres treated 10 days with recombinant Shh or control media.

In order to explore prostate stem cell self-renewal and differentiation directly, prostate primary cells from 10-week-old mice were isolated and subjected to 3D sphere culture in vitro (Supplementary Fig. 4). To test prostate stem cell self-renewal in the absence of YAP, prostate spheres were transfected with shYAP lentivirus and passaged. YAP ablation significantly inhibited the sphere formation potency of prostate stem cells, resulting in decreased sphere number and reduced sphere size (Fig. 8A–C). We also explored the expression of YAP and its direct target genes in cultured prostate spheres. The mRNA expression of YAP and its target genes were dramatically decreased in shYAP prostate spheres (Fig. 8D).

To further determine whether YAP modulates prostate stem cells through the Notch signaling and Hedgehog signaling, the mRNA expression of both pathway genes in cultured prostate spheres and scYAPKO prostates were examined. Notch1, a critical modulator of early prostate development, in shYAP prostate spheres and scYAPKO prostates was significantly reduced (Fig. 8E, F). Consistently, its target gene Hes1, as well as Dll1 were also decreased. Meanwhile, the expression of Shh and its target genes including Gli1, CyclinD1, and CyclinE1 was also reduced. Together, these results suggest that Notch signaling and Hedgehog signaling are regulated by YAP. To further confirm the above results, cultured prostate spheres were treated with Shh. Interestingly, Shh treatment significantly increased the size of shYAP prostate sphere, but it remained smaller in diameter than control prostate spheres (Fig. 8G). The results indicate that Shh treatment partially restored stem cell function in shYAP prostate spheres.

## Discussion

In this study, we elucidate the crucial role of YAP as a modulator of prostate stem cell, prostate development and regeneration via multiple models. YAP is highly expressed in mouse urogenital sinus-enriched prostate stem cell. YAP deletion on prostate stem cell attenuated prostate size, branching morphogenesis and luminal epithelial cell enfolding by diminishing cell proliferation. Simultaneously, YAP ablation on stem cell affected its cell fate determination leading to luminal cell maturation failure. Moreover, YAP inhibition significantly affected the regeneration process of mice prostate, leading to impaired regenerated prostate. Mechanistically, YAP regulates the expression of Notch and Hedgehog pathway genes, which are critical for prostate stem cell activity and prostate development.

Over the past two decades, YAP, as a key downstream component of the Hippo pathway, has increasingly been connected with developmental processes and tissue repair, being intimately related to the function of tissue-specific progenitor cells. Plenty of studies have suggested that YAP activity is key for the growth of whole organs, for amplification of tissue-specific progenitor cells and for cell proliferation. The simple overexpression of YAP in the liver is sufficient to induce a fourfold increase in liver mass caused by the proliferation of mature hepatocytes [26, 38]. On the other hand, liver-specific inactivation of YAP causes a decrease in hepatocyte proliferation and an increase in apoptosis [39]. Conditional deletion of YAP in embryonic cardiomyocytes affect their proliferation leading to severe hypoplasia, while overall heart size is increased via YAP overexpression [40–42]. YAP overexpression in transgenic mice intestine expands its progenitor cells and intestine cell proliferation [26]. Moreover, lack of YAP impairs intestine epithelia proliferation and crypt repopulation leading to rapid death during intestinal regeneration [43]. However, few studies focused on YAP function during prostate development and prostate stem cell. A previously study has reported that YAP deletion embryos arrested during developmental around E8.5 and displayed developmental perturbations that included a notably shortened body axis, convoluted anterior neuroepithelium, caudal dysgenesis, and failure of chorioallantoic fusion [24]. Since plenty researches have showed that YAP plays a key role in regulating stem cell, we applied YAP ablation on stem cells to investigate its function during prostate development. Therefore, we established a unique conditional knockout transgenic mouse model to investigate YAP function in prostate stem cell and development by inserting CreER recombinase behind the promoter of CD133 [44, 45].

As a male sex accessory gland, epithelial as well as mesenchyme AR and a series of common and organ-specific morphological regulatory genes expressed in unique temporal and spatial patterns can regulate various aspects of prostate development and regeneration [10, 34, 46–51]. Meanwhile, the delicate balance between stem cell differentiation and self-renewal drives major prostate development and regeneration. It has been reported that Lgr4/Gpr48 modulates prostate stem cell properties through Wnt/β-catenin signaling, and Lgr4 ablation mice had comprised branching morphogenesis and delayed epithelial differentiation, leading to decreased prostate size and impaired luminal cell function [15]. In our study, we employed YAP deletion specifically on stem cells rather than the whole genome and we surprisingly found that the phenotype of scYAPKO mouse prostate was similar to that of Lgr4 ablation mouse. In contrast, our data indicate that YAP modulates prostate stem cell via the Notch and Hedgehog pathways. Plenty of studies have highlighted the critical role of Notch signaling in prostate development and growth, and it has been reported that Notch1 inactivation leads to defects in prostate epithelial differentiation [52–54]. Dalrymple et al. demonstrated that Notch signaling is essential for the survival of transient amplifying prostate cells [55]. Furthermore, Shahi et al. found that the Notch signaling plays critical roles in prostate progenitor cells and concluded that Notch signaling is required to modulate the cell cycle and permit normal development and differentiation of prostate spheres [56]. Consistent with our results, Li et al. suggested that cell-autonomous epithelial Shh-Gli signaling and stromal Gli signaling are both essential to the renewal and differentiation potential of epithelial stem and progenitor cells [57, 58].

In conclusion, our data indicate that YAP plays crucial roles in normal prostate development, ductal branching and epithelial cell differentiation. Recently, a numerous of publication suggested dysregulation of YAP is associated with plenty of human cancer initiation and cancer stem cell proliferation, including prostate cancer [36, 59–62]. Nguyen et al. have found that YAP overexpression can induce the development of age-related prostate tumors [60]. Furthermore, Lee et al. reported that YAP overexpression contributes to the development of enzalutamide resistance by induction of cancer stemness [62]. Our recent studies also found that YAP was overexpressed in prostate cancer stem cell and YAP inhibition significantly diminished its sphere-forming ability in Matrigel (data not shown). Therefore, we proposed that YAP plays important roles not only in prostate stem cell but also in prostate cancer stem cell and tumor progression. Our findings argue that the investigation of roles for YAP in human prostate cancer is highly warranted from another perspective.

## Materials and methods

### Mice

All animal experiments were conducted in accordance with the principles and procedures of the Guiding Principles for the Care and Use of Animal Research and were approved by the Institutional Animal Care Committee. YAP^fl/fl^ (Stock Number: 027929) and CD133-CreER (Stock Number: 017743) mice were purchased from The Jackson Laboratory and described previously [27, 39]. Combining the conditional site-specific recombination system with tissue-specific expression of CreER allows gene modification in a spatiotemporally regulated manner. It is worth noting that the mode and dosage of Tamoxifen administration in late pregnancy is closely related to the pregnancy abortion rate of the female mice [44]. The study was once suspended due to the high abortion rate. After many attempts and failures, we finally found a suitable administration mode and dosage.

All mice were maintained on the C57BL/6 background. DNA extracted from mice tails was used for PCR to determine the genotype of the mice. Primers used for genotyping YAP^fl/fl^ and CD133-CreER mice are listed below:

**Table:** 

Primer	Sequence (5’-3’)	Product size (bp)
YAP-5loxp-F	TGAGGAGCTTTTAGCATTGGTGCAGT	Wild-type: 200
YAP-5loxp-R	AGCAGTGTGGTTACTTTTCCAGGTT	Mutant: 311
YAP-3loxp-F	AGCCTTTTTGCAGACTTTTGTGGCA	Wild-type: 305
YAP-3loxp-R	AACGTCATCTCTTCCCTAAGTCCCT	Mutant: 392
Common	CAGGCTGTTAGCTTGGGTTC	
Wild-type reverse	TGCTGATTGCCTTCTGTCTG	Wild-type: 586 bp
Mutant reverse	AGGCAAATTTTGGTGTACGG	Mutant: 320 bp

We defined midday of the day of vagina plug as embryonic day E0.5 and most pregnant mice give birth on E20 in our study. Also, induced Cre recombination, as previously described, were administered by Tamoxifen to chosen genotype dams at E17 [44]. Tamoxifen (T5648, Sigma) was dissolved at a concentration of 20 mg/ml in corn oil (C8267, Sigma) and first administered by intraperitoneal injection at the selected time points (6 mg/40 g of body weight), then changed to oral gavage (5 mg/40 g of body weight) due to the high abortion rate.

Castration of 10-week-old male mice was performed using standard protocols. Two weeks after castration, placebo pellets, DHT pellets (Innovative Research of America, Sarasota, FL, 7.5 mg) and Verteporfin (MedChemExpress, 1.5 mg/d/25 g of body weight) were implanted and administrated by intraperitoneal injection according to the experimental protocol. Following prostate regeneration, mice were sacrificed at different time points.

### Mouse prostate dissection, branching morphogenesis, and histology

Mice were euthanized, serum was obtained via cardiac puncture, and the whole urogenital tract was excised into PBS. Prostate lobes were dissected under an illumination microscope, weighted and further fixed in 4% paraformaldehyde for 6–8 h, depending on size. Fixed tissues were then serially dehydrated with ethanol, embedded in paraffin, and completely sectioned according to standard procedures. Sections of 5-µm thickness were stained with hematoxylin and eosin. ImageJ was applied for further image analysis.

Micro-dissections of prostate lobes were performed as previously described [9]. Distinct ductal networks of individual prostate lobe were observed after incubation in 1% collagenase-PBS at 37 °C for 30 min. Numbers of main ducts and distal ductal tips from three independent experiments were counted for statistical analysis.

### Immunohistochemistry and immunofluorescence

Sections were dewaxed in xylene and rehydrated in graded ethanol concentrations under standard procedures. The antigens were retrieved in 10 mM sodium citrate buffer (PH6.0) for 15 min at 95 °C. Then the slides were incubated in 3% H_2_O_2_ and 10% normal goat serum for 10 min individually to block endogenous peroxidase activity. Subsequently, primary antibodies were used to incubate with the slides overnight at 4 °C. The primary antibodies used were YAP (Abcam ab14074), AR (Abcam ab74272), Ki67 (Cell Signaling Technology 12202 S), CK5 (Abcam ab52635), CK14 (Proteintech Group 60320-1-Ig) and CK8 (Abcam ab9023 or ab53280). Then second antibody was added and incubated for 1 h. Development was achieved through use of 3^’^-3’-diaminobenzidene, and then Mayer’s hematoxylin was applied to counterstain the sections. For immunofluorescence, a fluorescein isothiocyanate-labeled secondary antibody (Abcam) was applied to slides. Tissue sections were then counterstained with 4’,6-diamidino-2-phenylinodole. For the detection of apoptosis, the Terminal-deoxynucleotidyl Transferase Mediated Nick End Labeling (TUNEL) Apoptosis Assay Kit (Beyotime Biotechnology) was used according to the manufacturer’s instructions.

### Co-immunoprecipitation (IP) and immunoblot analysis

Whole-cell lysates from tissue were incubated with lysis buffer (pH 7.4, 0.025 M Tris, 0.15 M NaCl, 0.001 M EDTA, 1% NP4 0, 5% glycerol, 5 mM PMSF) for total protein extraction. Co-IP was performed using Pierce™ Crosslink Magnetic IP/Co-IP Kit (Thermo Scientific) following the manufacture’s instructions. For each experiment, 10 µg YAP antibody (Cell Signal Technology 14074) was used. The immunoprecipitates were then subjected to SDS-PAGE and standard immunoblotting procedures. Pan-TEAD antibody (Cell Signal Technology 13295) was used.

### RNA extraction and quantitative RT-PCR

Total RNA was isolated from prostate tissue and primary prostate sphere using Trizol reagent (Invitrogen) and then applied to cDNA synthesis via the Reverse Transcription System (Thermo Scientific). PCR was performed using Applied Biosystems 7900 RT-PCR System (Thermo Scientific) and SYBR Green PCR Master Mix (Roche) to analyze mRNA expression. The relative expression of each gene was calculated using the comparative Ct method and normalized to GAPDH. Primer sequences are listed in the Supplementary Table.

### Prostate sphere-forming assay and in vivo prostate regeneration

Prostate primary cell isolation in 8-week-old mice and prostate sphere-forming assay were performed as described [63–65]. After 8 days of culture, prostate spheres were collected, counted, and passaged by dispase digestion. Approximately 15,000 cells were then reseeded in Matrigel in a 12-well plate for secondary spheroid formation. In several experiments, 0.25ug/ml recombinant mouse Sonic Hedgehog (MedChemExpress) was used to treat prostate spheres.

Sphere lentivirus infection was performed as described [65]. Briefly, shYAP lentivirus was transfected into sphere cells using the centrifugation method at 2000 rpm and room temperature for 90 min. Then the pellet was resuspended and reseeded as described above [63].

In vivo prostate regeneration was performed as described [63, 64]. In brief, UGSM cells were isolated and cultured as described. Then infected or control sphere cells were mixed with UGSM cells and resuspended in Matrigel on ice. The cell mixture was injected subcutaneously into SCID mice. The transplants can be harvested for further analysis after 2 months.

### Statistical analysis

Statistical analysis was performed with GraphPad Prism 8.0 software (San Diego, CA, USA). Differences between experiment and control group were measured using either unpaired two-tailed Student’ *t* test or one-way ANOVA. *P* < 0.05 was considered statistically significant. *P < 0.05, **P < 0.01. The results were shown as mean ± SEM.

### Supplementary information

Supplentary file

Original Data File

## Data Availability

All datasets generated and analyzed during this study are included in this published article and its Supplementary Information files. Additional data are available from the corresponding author upon reasonable request.
