# Supplementary material for: YAP is required for prostate development, regeneration, and prostate stem cell function
Source: Cell Death Discov. 2023 Sep 9;9:339. doi: 10.1038/s41420-023-01637-1 (PMC10492789; doi:10.1038/s41420-023-01637-1)
Supplement: Supplementary file 1 — Supplentary file [file 41420_2023_1637_MOESM1_ESM.docx]

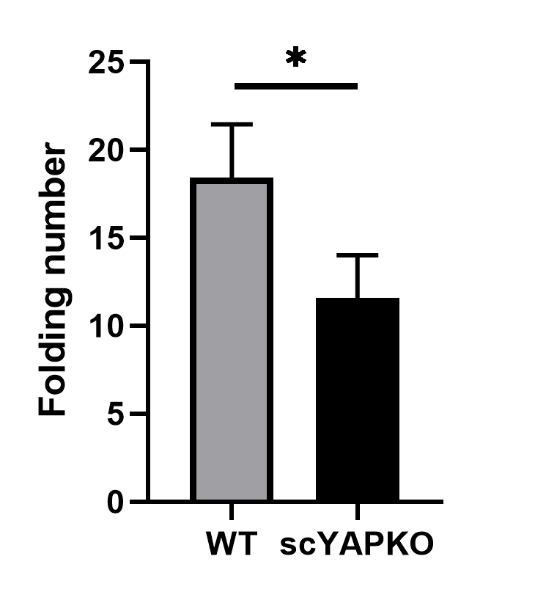


Supplemental Figure 1. The relative prostate folding number of scYAPKO and WT mouse prostates was quantified. Bar graph expresses mean ± SEM from 3 pairs of mice, 5 section per prostate, P<0.05.


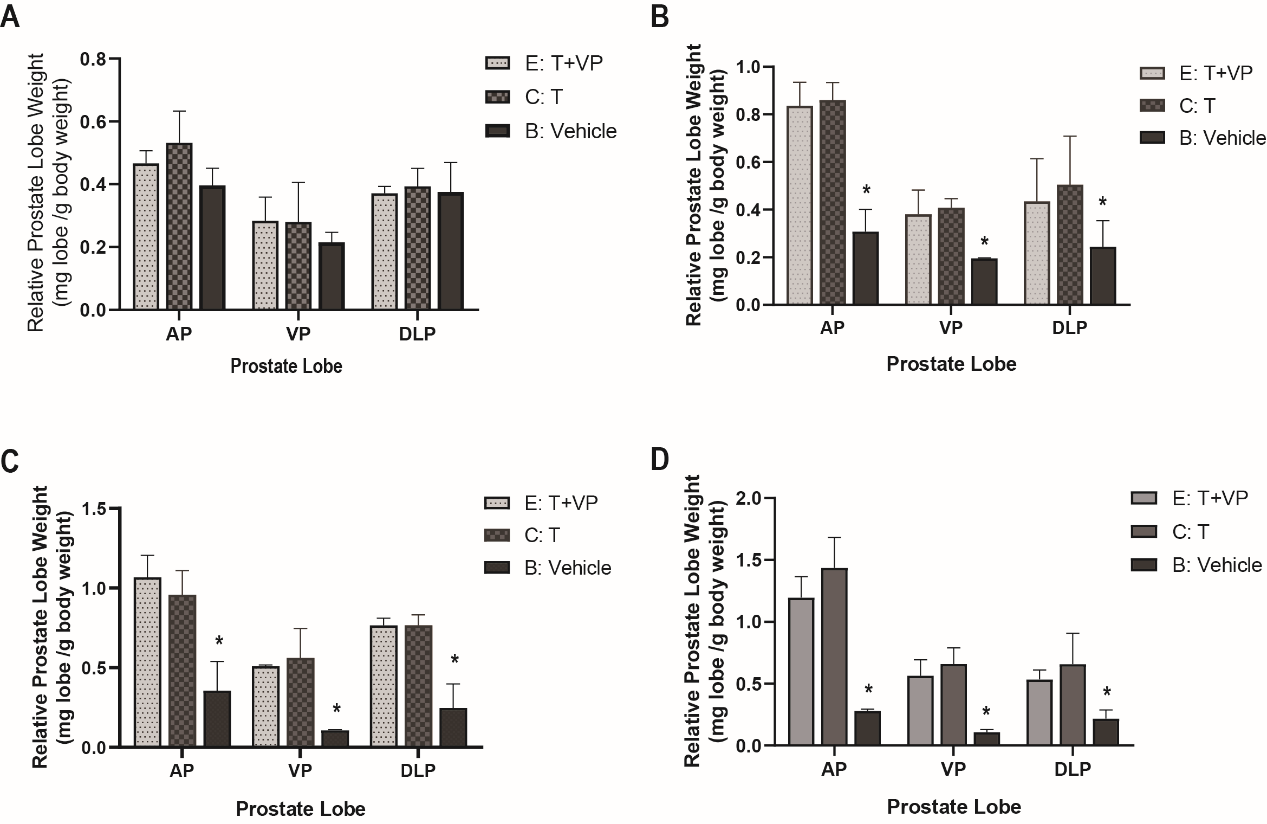


Supplemental Figure 2. The relative prostate lobe weight (mg lobe/g body weight) of regenerated mice at consecutive time points (A. 1d; B. 3d; C.7d; D. 14d) were measured.


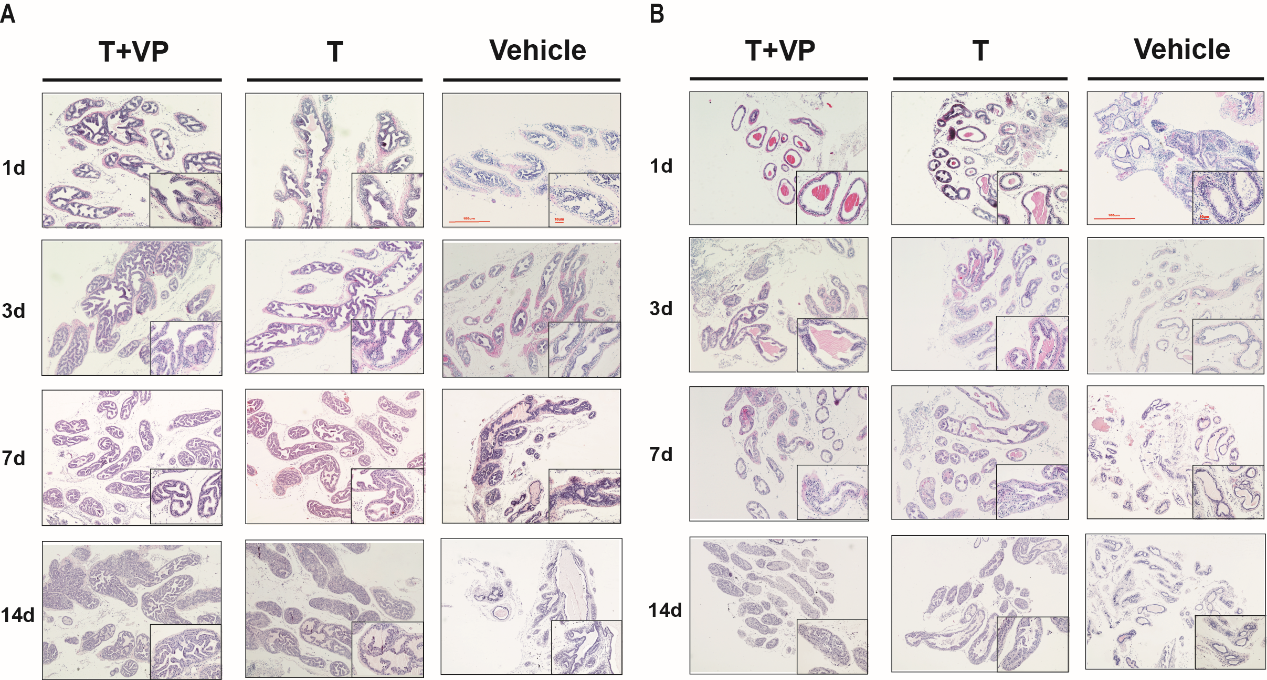


Supplemental Figure 3. Hematoxylin & Eosin staining of anterior (A) and dorsal lateral (B) prostate sections at indicated regeneration stages.


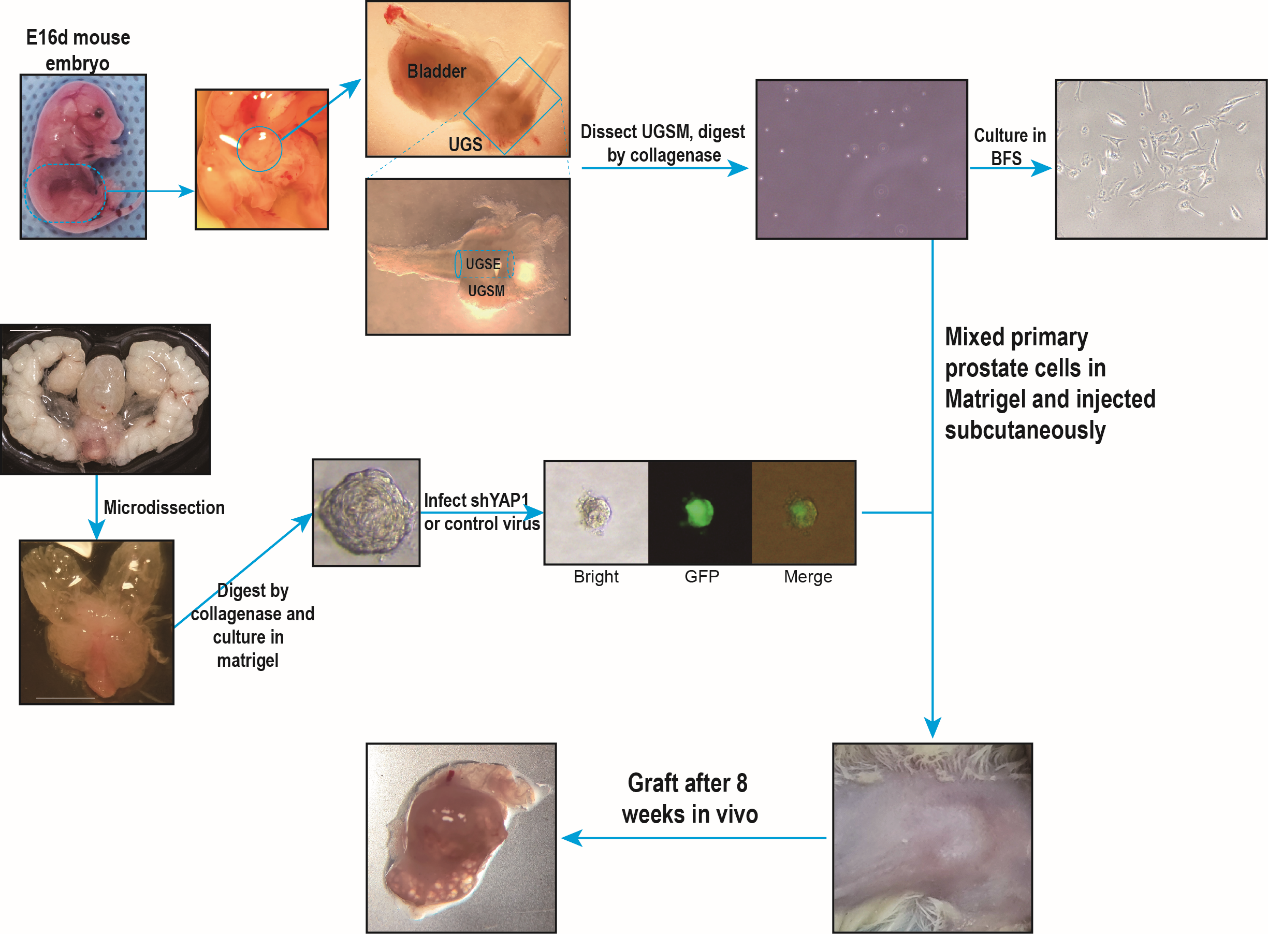


Supplemental Figure 4. Outline of the prostate sphere culture and prostate regeneration system.

Supplementary Table. Primer sequences for real-time PCR.

| **Primer** | **Sequence** |
| --- | --- |
| mYAP1-F | AGACGACTTCCTCAACAGTGT |
| mYAP1-R | TCTCCTTCCAGTGTGCCAAG |
| mTEAD4-F | ATGTCGTCTGCACAGATCGTC |
| mTEAD4-R | TTGCCAAAACCCTGAGATTGC |
| mCTGF-F | CCGAGTTACCAATGACAATACC |
| mCTGF-R | CTTGACAGGCTTGGCGATT |
| mCyr61-F | CTGCGCTAAACAACTCAACGA |
| mCyr61-R | GCAGATCCCTTTCAGAGCGG |
| mSOX2-F | ACCAGCTCGCAGACCTACAT |
| mSOX2-R | GCCTCGGACTTGACCACAGA |
| mNanog-F | AACTCTCCTCCATTCTGAACCT |
| mNanog-R | GACCATTGCTAGTCTTCAACCA |
| mAR-F | GCCTCCGAAGTGTGGTATCC |
| mAR-R | CCTGGTACTGTCCAAACGCA |
| mKLK3-F | GGGGCTACACGAAGGAACAA |
| mKLK3-R | GCCACCGGAATCTCCCTTAC |
| mProbasin-F | GTGTGGGAGGAGATGCAACC |
| mProbasin-R | TGCTATGCTCCCTTCATACTGT |
| mTGF-β-F | CTGCTGACCCCCACTGATAC |
| mTGF-β-R | GGGGCTGATCCCGTTGATT |
| mFKBP5-F | AGCAACGGTAAAAGTCCACCT |
| mFKBP5-R | TGCATCTTCACCAGGGCTTT |
| mNkx3.1-F | GTCTTGGAGAAGAACTCACCATTG |
| mNkx3.1-R | GCCAACCTGCCTCAATCACTAAGG |
| mNotch1-F | CACAGAAGCGAGGCATTGAC |
| mNotch1-R | GGTTAGGTGAGCACTCGTCC |
| mJag1-F | CTACATAGCCTGTGAGCCTTCC |
| mJag1-R | GCAACCGCAGCAATAAGTGAG |
| mHes1-F | CAACACGACACCGGACAAAC |
| mHes1-R | GGAATGCCGGGAGCTATCTT |
| mDll-F | TCAATGGAGGACGATGTTCAGA |
| mDll-R | CAGGTAAGAGTTGCCGAGGT |
| mShh-F | TCCCAACGTAGCCGAGAAGA |
| mShh-R | TTGTCTTTGCACCTCTGAGTC |
| mGli1-F | CAGCATGGGAACAGAAGGACT |
| mGli1-R | GAGAGAGCCCGCTTCTTTGT |
| mGli2-F | ACTTTTGTCTCCTCGGGTCC |
| mGli2-R | AGGACAGGCCTTTTTCCCTG |
| mGli3-F | GATTCCTTTGAGAAACAAGCTGA |
| mGli3-R | CCAGGACTTTCATCCTCATTGGA |
| mCycE-F | GACACAGCTTCGGGTCTGAG |
| mCycE-R | TTCTGGAGCGGACTGAAAGG |
| mCyclinD-F | GGATGCTGGAGGTCTGTGAG |
| mCyclinD-R | GCAGGCGGCTCTTCTTCAA |
| mcMyc-F | CGTTGGAAACCCCGCAGAC |
| mcMyc-R | GCTCTGCTGTTGCTGGTGATA |
| mSmo-F | AGCCTTTGCGCTACAACGTG |
| mSmo-R | TTCCGGAGGCCGGACCA |
| mBmp4-F | TCCGTCCCTGATGGGATTCT |
| mBmp4-R | GGAATCATGGTGTCTCATTGGT |
| mBmp7-F | GCTCCAAGACGCCAAAGAAC |
| mBmp7-R | GTGCAATGATCCAGTCCTGC |
| mSnail-F | CAGGACGCGTGTGTGGAGTT |
| mSnail-R | ACTTGGGGTACCAGGAGAGAG |
| mSMAD4-F | TAATCGCGCATCAACGGAGA |
| mSMAD4-R | TTGTGAACTGGCCTTGTGGA |
| mWnt4-F | CGAGCAATTGGCTGTACCTGG |
| mWnt4-R | GCACTGAGTCCATCACCTCAA |
| mWnt5a-F | CTTGTTGCTCCGGCCCAG |
| mWnt5a-R | GGGTTATTCATACCTAGAGACCACC |
| mWnt7b-F | CTAGGAAGGCCAGTGACCAGA |
| mWnt7b-R | CCACGGATGACAATGCTCTGTAAG |
| mAxin2-F | GGTCCTGGCAACTCAGTAACA |
| mAxin2-R | CTCATGTGAGCCTCCTCTCTTTT |
| mb-catenin-F | ACAAGCCACAGGATTACAAGAA |
| mb-catenin-R | CACCAATGTCCAGTCCAAGAT |
| mCD44 F | ACCTTGGCCACCACTCCTAAT |
| mCD44 R | ATGGTTGTTGTGGGCCGAA |
