# Supplementary figures and images for: YAP is required for prostate development, regeneration, and prostate stem cell function
Source: Cell Death Discov. 2023 Sep 9;9:339. doi: 10.1038/s41420-023-01637-1 (PMC10492789; doi:10.1038/s41420-023-01637-1)

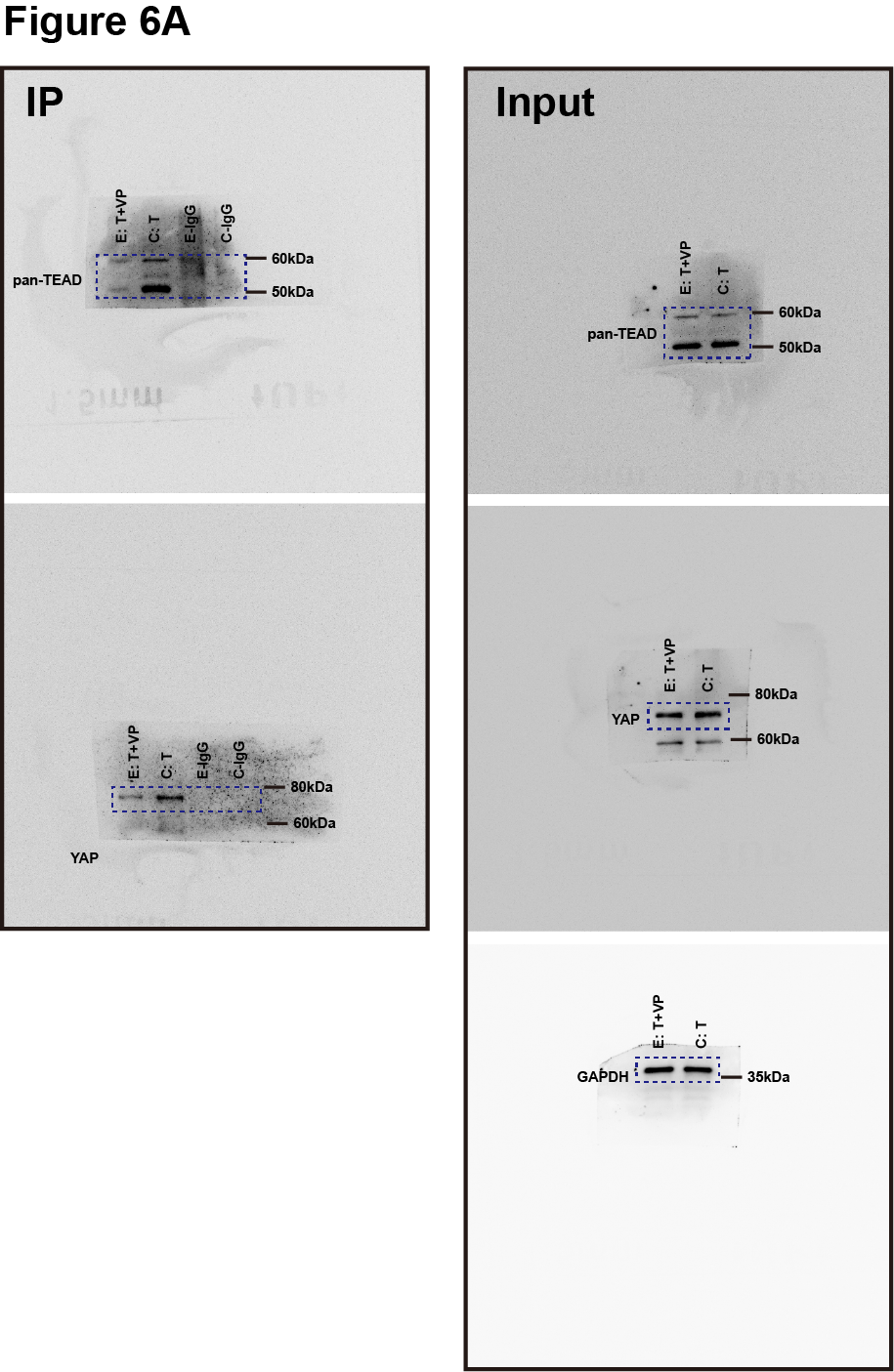

Supplement: Supplementary file 2 — Original Data File [file 41420_2023_1637_MOESM2_ESM.docx]
